# Comparative performance characteristics of the urine lipoarabinomannan strip test and sputum smear microscopy in hospitalized HIV-infected patients with suspected tuberculosis in Harare, Zimbabwe

**DOI:** 10.1186/s12879-016-1339-z

**Published:** 2016-01-22

**Authors:** Lynn Sodai Zijenah, Gerard Kadzirange, Tsitsi Bandason, Maria Mary Chipiti, Bevel Gwambiwa, Forget Makoga, Pauline Chungu, Philip Kaguru, Keertan Dheda

**Affiliations:** 1 Departments of Immunology, University of Zimbabwe College of Health Sciences, Harare, Zimbabwe; 2 Departments of Medicine, University of Zimbabwe College of Health Sciences, Harare, Zimbabwe; 3 Biomedical Research and Training Institute, Harare, Zimbabwe; 4 Lung Infection and Immunity Unit, Division of Pulmonology, Department of Medicine, University of Cape Town, Cape Town, South Africa

**Keywords:** Urine LAM strip test, Sputum smear microscopy, Hospitalized HIV-infected patients, CD4 counts

## Abstract

**Background:**

In Zimbabwe, sputum smear microscopy (SSM) is the routinely used TB diagnostic tool in hospitalised HIV-infected patients. However, SSM has poor sensitivity in HIV-infected patients. We compared performance of urine lipoarabinomannan strip test (LAM) and SSM among hospitalized HIV-infected patients with suspected TB.

**Methods:**

Hospitalized HIV-infected patients with suspected TB were randomized to LAM plus SSM or SSM alone groups as part of a larger multi-country parent study. Here we present a comparison of LAM versus SSM performance from the Zimbabwe study site. LAM analyses (grade 2 cut-off) were conducted using (i) a microbiological reference standard (MRS; culture positivity for *M.tb* and designated definite TB) and (ii) a composite reference standard (CRS; definite TB plus probable TB i.e. patients with clinical TB excluded from the culture negative group). CRS constituted the primary analysis.

**Results:**

82/457 (18 %) of the patients randomized to the LAM group were *M.tuberculosis* culture positive. Using CRS, sensitivity (%, 95 % CI) of LAM was significantly higher than SSM [49.2 (42.1-56.4) versus 29.4(23.2-36.3); *p* < 0.001]. Specificity and PPV were 98.1 %, and 95.8 %, respectively. By contrast, using MRS, LAM sensitivity was similar to SSM and specificity was significantly lower, however, the combined sensitivity of LAM and SSM was significantly higher than that of SSM alone, *p* = 0.009. Using CRS, LAM sensitivity (%, CI) was CD4 count dependent [60.6(50.7-69.8) at ≤50 cells/μL; 40.0(22.7-59.4) at 51-100 cells/μL, and 32.8(21.0-46.3) at >100 cells/μL. The combined sensitivity of LAM and SSM was higher than SSM alone being highest at CD4 counts <50 cells/μL [67.6(57.9-76.3); *p* = <0.001]. Specificity of LAM or SSM alone, or of combined LAM and SSM was >97 % in all the 3 CD4 strata.

**Conclusion:**

Among hospitalized HIV-infected patients with suspected TB, the sensitivity of LAM is significantly higher than that of SSM, especially at low CD4 counts. LAM and SSM are complimentary tests for diagnosis of TB in HIV-infected patients. We recommend a combination of LAM and SSM for TB diagnosis in HIV-infected patients with low CD4 counts in HIV/TB co-endemic countries, where alternative methods are unavailable.

## Background

Tuberculosis (TB) is the major cause of morbidity and mortality among HIV-infected patients in southern Africa which bears 80 % of the global HIV burden [1]. Sputum smear microscopy (SSM), the routinely used microbiological TB diagnostic tool in Zimbabwe, has poor sensitivity (20–50 %) identifying only a minority of HIV/TB co-infected patients [2]. The ability of SSM to identify patients with TB may rely on the patient’s ability to produce “quality sputum”. Studies have shown that HIV infected patients are not able to produce “quality sputum” or produce paucibacillary sputum resulting in possibly false negative microscopy results [3].

Alere has developed a point of care (POC) lateral flow urine Lipoarabinomannan strip test (LAM) which has been evaluated in various countries [4–10] with widely varying performance characteristics. The current consensus is that the test is most suitable for HIV-infected patients with CD4 counts <200 cells/μl [11]. However, there is lack of clarity about the context in which LAM testing should be used. In the current study we aimed to compare the performance characteristics of the LAM and SSM among hospitalized HIV-infected patients using culture as the reference standard.

## Methods

### Ethical approval

The study was approved by the Medical Research Council of Zimbabwe (MRCZ/A/1680). All participants gave their written informed consents.

### Study design and study participants

The original study was a multi-centric randomized controlled trial (RCT) registered with Clinicaltrials.gov number:NCT01770730.

In the RCT, hospitalized HIV-infected patients with suspected TB were randomized to LAM group where, LAM, SSM, chest x-ray and culture were performed or no LAM group, where SSM, chest x-ray and culture were performed, using a computer generated allocation list. Patients were followed-up at 2 months. We analysed data from patients randomized to the LAM group only where both LAM and SSM were performed on the same patient. Participants’ demographics medical examinations, TB symptoms were recorded.

At enrolment, at least two sputum specimens were collected from each participant for same day microscopy. One sputum specimen was submitted for culture and a third sample if available was stored at -80 °C. Blood was collected for HIV testing and CD4 count. Spot urine was collected for LAM.

### Bacteriological testing

SSM using a fluorescence microscope was done with auramine stain for screening and confirmation of auramine positive smear was with Ziel-Neelsen (Z-N) stain.

LAM was performed by a State certified laboratory technologist according to manufacturer’s instructions. Briefly 60 μL of freshly collected urine was applied to the test strip, incubated at room temperature for 25 min and the result recorded as negative if there was no presence of any band or recorded as positive and band graded as 1 or 2 or 3, 4 or 5 using the manufacturer’s reference card with bands of graded intensity. The analysis was conducted using grade ≥2 cut-off, which has been reported to have better inter-observer reliability and good rule-in value in hospitalized patients [4]. However, it is important to note that the grade 2 cut-point considered positive in this study using the pre-January 2014 reference card corresponds to the first positive band (grade 1) in the current post-January 2014 reference card [12]. The Mycobacteria Growth Indicator Tube (MGIT BD Microbiology Systems, Cockeysville, MD, USA) culture was performed at a central laboratory on sputum decontaminated using 4 % NaOH. Suspected positive cultures were confirmed using Z-N staining. MPT64 antigen detection was used for speciation of MGIT positive cultures and by growth at different temperatures if antigen detection was negative [13].

### Definitions of the microbiological and composite reference standards

MGIT which is routinely used as the reference TB diagnostic tool has limitations in HIV-infected patients particularly those with advanced Acquired Immunodeficiency Syndrome (AIDS), low sputum bacillary load who are sputum scarce. The following definitions were used to categorise patients into diagnostic groups based on smear microscopy, culture and empirical treatment (radiological findings and/or clinical symptoms) coupled with response to treatment at the 2-months follow-up:

### Definite TB

MGIT *Mycobacterium Tuberculosis* (MTB) positive.

### Probable TB

MGIT MTB negative, a clinical and/or radiological finding highly suggestive of active TB and supported by response to anti-TB treatment at the 2-month follow-up.

### Non-TB

No evidence of MTB and an alternative diagnosis made and thus not treated for TB. Patients who were MTB culture negative but were commenced on anti-TB treatment empirically, yet showed no response to treatment at the 2-months follow-up were also included in this group. Patients who were culture positive for non-tuberculosis mycobacteria and were not receiving anti-TB treatment were also included in this Non-TB group.

We conducted the comparative performance of LAM using grade 2 cut cut-off positive results and SSM using a (i) MRS (utilizing definite TB culture positive versus culture negative) and (ii) CRS (utilizing definite TB plus probable TB and patients with clinical TB excluded from the culture negative group). Using the CRS, sensitivity was calculated using the combined definite TB and probable TB whilst the specificity calculations were based on non-TB definitions

### Data capture and analysis

The clinic and laboratory data was entered by two dedicated data entry staff into a MS Access database. Epidata software was used to validate dual entry of the data.

Demographic, clinical and microbiological characteristics of different patient sub-groups were compared using Chi-squared test and Wilcoxon rank-sum test as appropriate. For comparison of diagnostic tests results, MTB species identification was used as reference standard for culture positivity. Sensitivity, Specificity, Negative Predictive Value (NPV), Positive Predictive Value (PPV) were calculated for all diagnostic tests (with 95 % confidence intervals). All statistical tests were considered significant at *p* = 0.05. STATA Version 12 (Stata Corp, Texas, USA) was used for all statistical analyses.

## Results

### Patient recruitment

**Fig. 1:**
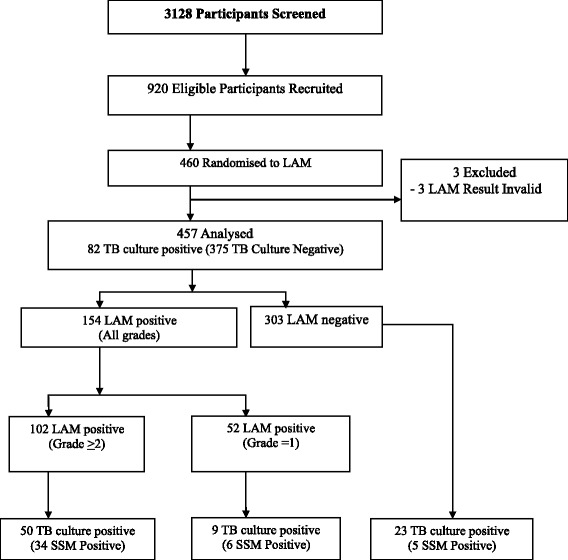
Participants Flow. Flow chart of study participants and analysis. LAM = urine Lipoarabinomannan strip test; SSM = sputum smear microscopy

The flow chart of the study population is outlined in Fig. 1. Of the 3128 hospitalized HIV-infected patients screened, 920 with suspected TB were enrolled between 07 January 2013 and 26 September 2014 with 460 randomly assigned to LAM and 460 to No LAM. Three out of 460 (0.007 %) had invalid LAM results and were thus excluded from the analysis. The baseline demographics and clinical characteristics of the participants in the LAM group based on TB diagnosis definition are shown in Table 1. 82 /457 (18 %) had definite TB, 115/457 (25 %) had probable TB, 260/457 (57 %) were classified as non-TB. The median CD4 count for non-TB, 71 cells/μL, IQR: 24–180 was significantly lower than that of the combined definite TB; 41 cells/μL, IQR: 15-88 and probable TB, 43 cells/μL, IQR: 17-128 groups; *p* < 0.0001. The proportion of patients with fever was significantly higher in the combined probable TB and definite TB groups, *p* = 0.0001. A significantly higher proportion of patients on anti-TB treatment with definite TB combined with probable TB, reported improved TB symptoms at the 2-months follow-up when compared to the non-TB patients, *p* = 0.003.

**Table 1:** Demographics, clinical and microbiological characteristics of study patients stratified by TB diagnostic group

	All	Definite TB	Probable TB	Non-TB	*P*-value
	*N* = 457	*N* = 82	*N* = 115	*N* = 260	
Demographics					
Median age (IQR)	37	36	35	38	NS
	(31-44)	(32-41)	(29-44)	(31-45)	
Female (%)	241 (53)	41 (50)	63 (55)	123 (52)	NS
Median CD4 count (cells/μL, IQR)	55	41^a^	43 ^a^	71*	<0.0001
	(18-153)	(15-88)	(17-128)	(24-180)	
Previous TB (%)	95 (21)	12(15)	26(23)	57(22)	NS
Clinical features					
Cough >2 wks (%)	446 (98)	82(100)	113(98)	251(97)	NS
Drenching night sweats (%)	299 (65)	51(62)	81(70)	167(56)	NS
Weight loss (%)	361 (79)	68(83)	92(80)	201(77)	NS
Fever > 38 °C (%)	334 (73)	68 (82)^a^	90 (78)^a^	176(68)*	<0.005
CXR compatible with TB (%)	252 (55)	61(74)	76(66)	115(44)	<0.0001
Commenced on TB Treatment (%)	218 (48)	81(99)^a^	115(100)^a^	22(8)*	<0.0001
TB treatment based on:	*n* = 218	*n* = 81	*n* = 115	*n* = 22	
Empirically (%)	87(40)	11(14)^a^	60(52)^a^	16(73)*	<0.0001
LAM positive grades 1 and 2^b^	104^b^ (48)	50(62)*	48(42)*	6(27)*	<0.005
SSM	16(7)	11(14)*	5(4)*	0(0)	<0.005
Culture	11(5)	9(11)*	2(2)*	0(0)	<0.005
TB symptoms at 2 months for those on treatment	*n* = 218	*n* = 81	*n* = 115	*n* = 22	
Improved TB symptoms on treatment at 2 month follow-up	137(63)	54(66)*	83(72)*	0(0)	0.003
Died of TB before 2 months follow-up	61(28)	19(23)^a^	32(28)^a^	10(45)	0.023
Lost to follow-up on treatment	2(1)	1(1)	0(0)	1(5)	NS

*CXR* Chest x-ray, *LAM* urine Lipoarabinomannan strip test, *SSM* sputum smear microscopy

*P*-values indicate significant differences between patient groups (marked with * to indicate comparison group) for different patients characteristics

* - Significantly different (*p* ≤ 0.05). ^a^ - Significant difference after combining two groups

*NS* Not significant (*p* > 0.05). ^b^102 patients had LAM ≥2 positivity and 2 had grade 1 and attending clinicians insisted on commencing anti-TB treatment for these 2 patients

### Comparative performance of SSM and LAM using MRS

Preliminary analysis showed that LAM grade ≥1 cut-off had a significantly higher sensitivity 72.0 %, CI: 60.9–81.3 versus SSM 54.9 %, CI: 43.5-65.9; *p* = 0.022. However, the specificity of LAM grade ≥1 cut-off was poor and significantly lower, 74.7 %, CI: 69.9–79.0 vs 95.7. CI: 93.2–97.5; *p* < 0.001. There was no significant difference in sensitivity between LAM grade ≥1 cut-off; 72 %, CI: 60.9–81.3) vs LAM grade ≥2 cut-off, 61 %, 49.6-71.6; *p* = 0.132. However, the specificity of LAM grade ≥2 cut-off, 86.1 %, CI: 82.2–89.5 vs LAM grade ≥1, 74.7 %, CI: 69.9–79.0; *p* < 0.001 was significantly higher.

All subsequent analysis reported in this study are based on LAM grade ≥ 2 cut-off.

Of the 457 patients, 82/457 (18 %) were culture positive. Using the MRS, there was no significant difference in sensitivity of LAM, 61.0 %, and SM; 54.9 %, *p* = 0.429 (Table 2). Specificity of SSM, 95.7 % was significantly higher than that of LAM; 86.1 %, *p* < 0.001. Similarly the PPV of SSM, 73.8 % was significantly higher than that of LAM; 49.0 %, *p* = 0.007 (Table 2). However, there was no significant difference in the NPV of SSM; 90.7 % and LAM; 91.0 % *p* = 0.876.

**Table 2:** Comparative performance of SSM versus LAM, and the combination of LAM and SSM versus SSM alone using the microbiological reference standard

	SSM versus LAM			Combined SSM and LAM versus SSM alone			
*N* = 457	SSM only	LAM only	*p*-value	SSM only	LAM only	SSM plus LAM	*p*-value
Sensitivity	54.9 %	61.0 %	0.429	54.9 %*	61.0 %	74.4 %*	0.009
(95 % CI)	(43.5–65.9)	(49.6-71.6)		(43.5-65.9)	(49.6–71.6)	(63.6-83.4)	
n + ve/total	45/82	50/82		45/82	50/82	61/825	
Specificity	95.7 %	86.1 %	<0.001	95.7 %*	86.1 %	84.8 %*	<0.001
(95 % CI)	(93.2-97.5)	(82.2-89.5)		(93.2-97.5)	(82.2-89.5)	(80.8-88.3)	
n -ve/total	359/375	323/375		359/375	323/375	318/375	
PPV	73.8 %	49.0	0.007	73.8 %*	49.0	51.7 %*	0.015
(95 % CI)	(60.9–84.2)	(39.0-59.1)		(60.9-84.2)	(39.0–59.1)	(42.3-61.0)	
NPV	90.7 %	91.0	0.876	90.7 %	91.0	93.8 %	>0.05
(95 % CI)	(87.6-93.6)	(87.5-93.8)		(87.6–93.6)	(87.5-93.8)	(90.7-96.1)	

*SSM* smear microscopy, *LAM* Urine Lipoarabinomannan strip test, *n + ve* number positive, *n –ve* number negative

*P*-value indicate significant differences between patient groups (marked with * and number to indicate comparison group) * - Significantly Different (*p* ≤ 0.05)

The combined sensitivity of SSM and LAM, 74.4 %, was significantly higher than that of SSM alone; 54.9 %, *p* = 0.009. Conversely, the combined specificity was lower than that of SSM alone, 84.8 % versus 95.7 %, *p* < 0.001, so was the combined PPV; 51.7 % vs 73.8 %, *p* = 0.015. The combined NPV did not differ from that of either test alone, *p* > 0.05 (Table 2).

### Comparative performance of SSM and LAM using CRS

In sharp contrast to MRS-based analysis, when the CRS was employed, the sensitivity of LAM, 49.2 % was significantly higher than that of SSM, 29.4 %; *p* < 0.001. The PPV for LAM and SSM (95.1 %) were similar, *p* = 0.996. However the NPV of LAM 71.8 % was significantly higher than that of SSM, 64.9 %; *p* = 0.042 (Table 3).

**Table 3:** Comparative performance of SSM versus LAM; and the combination of SSM and LAM versus SSM alone using composite reference standard

	SSM versus LAM			Combined SSM and LAM versus SSM alone			
*N* = 457	SSM only	LAM only	*p*-value	SSM only	LAM only	SSM plus LAM	*p*-value
Sensitivity	29.4 %	49.2 %	<0.001	29.4 %*	49.2 %	57.4 % *	<0.001
(95 % CI)	(23.2-36.3)	(42.1-56.4)		(23.2-36.3)	(42.1-56.4)	(50.1-64.4)	
n + ve/total	58/197	97/197		58/197	97/197	113/197	
Specificity	98.8 %	98.1 %	0.476	98.8 %	98.1 %	98.1 %	>0.05
(95 % CI)	(96.7-99.8)	(95.6-99.4)		(96.7-99.8)	(95.6-99.4)	(95.6-99.4)	
n -ve/total	257/260	255/260		255/260	255/260	255/260	
PPV	95.1 %	95.1 %	0.996	95.1 %	95.1 %	95.8 %	>0.05
(95 % CI)	(86.3-99.0)	(88.9-98.4)		(86.3-99.0)	(88.9–98.4)	(90.4-98.6)	
NPV	64.9 %	71.8 %	0.042	64.9 %*	71.8 %	75.2 %*	<0.001
(95 % CI)	(60.0-69.6)	(66.8–76.5)		(60.0-69.6)	(66.8-76.5)	(70.3-79.7)	

*SSM* sputum smear microscopy, *LAM* urine Lipoarabinomannan strip test, *n + ve* number positive, *n –ve* number negative

*P*-value indicate significant differences between patient groups (marked with * and number to indicate comparison group) * - Significantly different (*p* ≤ 0.05)

The sensitivity of SSM combined with LAM was significantly higher than that of SSM alone 57.4.8 %; *p* < 0.001. However there was no significant difference in combined specificity (>98 %), *p* > 0.05, nor PPV (>95 %); *p* > 0.05. The NVP of SSM combined with LAM was significantly higher (75.2 %0; *p* < 0.001 than SSM alone. (Table 3).

### Comparative performance of LAM and SSM stratified by CD4 count using MRS

The sensitivity of LAM versus SSM was not significantly different at CD4 counts: ≤50 cells/μL; 51-100 cells/μL and >100 cells/μL (Table 4). Specificity of SSM was significantly higher at all the three CD4 count strata compared to LAM (Table 4).

**Table 4:** Comparative performance of SSM versus LAM; and the combination of SSM and LAM versus SSM alone using the microbiological reference standard, stratified by CD4 count

*N* = 457	SSM versus LAM			Combined SSM with LAM versus SSM alone			
	SSM only	LAM only	*p*-value	SSM only	LAM only	SSM plus LAM	*p*-value
Sensitivity (95 % CI)							
CD4 (cells/μL)							
≤50	63.8 (48.5-77.3)	76.6 (62.0-87.7)	0.176	63.8 (48.5-77.3)*	76.6 (62.0-87.7)	87.2 (74.3-95.2)*	0.008
51-100	50.0 (24.70- 75.3)	43.8 (19.8-70.1)	0.723	50.0 (24.70- 75.3)	43.8 (19.8-70.1)	62.5 (35.4-84.8)	>0.05
>100	36.8 (16.3-61.6)	36.8 (16.3- 61.6)	-	36.8 (16.3-61.6)	36.8 (16.3- 61.6)	52.6 (28.9-75.6)	>0.05
Specificity (95 % CI)							
CD4 (cells/μL)							
≤50	93.0 (88.1-96.3)	80.7 (74.0-86.8)	0.001	93.0 (88.1-96.3)*	80.7 (74.0-86.8)	78.9 (72.1-84.8)*	0.001
51-100	98.4 (91.2-100.0)	90.2 (79.8-96.3)	0.052	98.4 (91.2-100.0)	90.2 (79.8-96.3)	90.2 (79.8-96.3)	>0.05
>100	97.9 (94.0-99.6)	90.9 (85.0-95.1)	0.010	97.9 (94.0-99.6)*	90.9 (85.0-95.1)	89.5 (83.3-94.0)*	0.004

*SSM* sputum smear microscopy, *LAM* Urine Lipoarabinomannan strip test

*P*-value indicate significant differences between patient groups (marked with * and number to indicate comparison group) * - Significantly Different (*p* ≤ 0.05) NS – Not Significantly Different (*p* > 0.05)

When combined, the sensitivity of SSM and LAM was highest at CD4 count ≤50 cells/μL and was significant higher than that of SSM alone; 87.2 %, CI: 74.3-95.2 *p* = 0.008. The specificity at the two CD4 count strata (≤50 cells/μL and >100 cells/ μL) for the combined SSM and LAM was significantly lower for SSM (Table 4).

### Comparative performance of LAM and SSM stratified by CD4 count using CRS

**Fig. 2:**
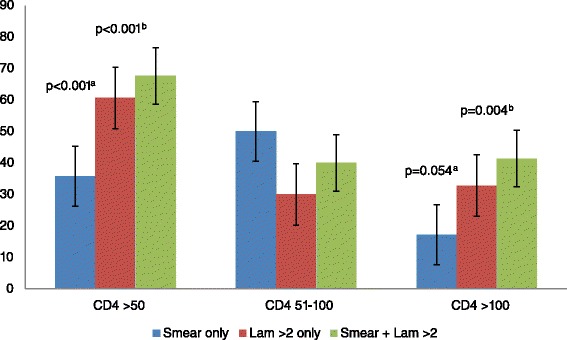
Comparative sensitivity of SSM versus LAM; and the combination of SSM and LAM versus SSM alone using composite reference standard stratified by CD4 counts. SSM; sputum smear microscopy; LAM; urine Lipoarabinomannan strip test. ^a^ P-value indicates significant differences between SSM only and LAM only. ^b^P-value indicates significant differences between SSM only and SSM plus LAM

The sensitivity of LAM was significantly higher at CD4 count ≤50 cells/μL; 60.6, 95 % CI: 50.7–69.8 versus SSM; 35.8, 95 % CI: 26.8–45.5, *p* < 0.001 (Table 5, Fig. 2). There was no significant difference in sensitivity at CD4 counts 51–100 cells/μL, *p* = 0.417. The specificity of SSM and LAM was not significantly different at the three CD4 counts strata (Table 5).

**Table 5:** Comparative performance of SSM versus LAM; and the combination of SSM and LAM versus SSM alone using composite reference standard, stratified by CD4 count

*N* = 457	SSM versus LAM			Combined SSM with LAM versus SSM alone			
	SSM only	LAM only	*p*-value	SM only	LAM only	SM plus LAM	*p*-value
Sensitivity (95 % CI)							
CD4 (cells/μL)							
≤50	35.8 (26.8-45.5)	60.6 (50.7-69.8)	<0.001	35.8 (26.8-45.5)*	60.6 (50.7-69.8)	67.6 (57.9-76.3)*	<0.001
51-100	30.0 (14.7-49.4)	40.0 (22.7-59.4)	0.417	30.0 (14.7-49.4)	40.0 (22.7-59.4)	50.0 (31.3-68.7)	>0.05
>100	17.2 (8.6-29.4)	32.8 (21.0-46.3)	0.054	17.2 (8.6-29.4)*	32.8 (21.0-46.3)	41.4 (28.6-55.1)*	0.004
Specificity (95 % CI)							
CD4 (cells/μL)							
≤50	97.2 (92.2-99.4)	97.2 (92.2-99.4)	>0.05	97.2 (92.2-99.4)	97.2 (92.2-99.4)	97.2 (92.2-99.4)	>0.05
51-100	100.0 (92.5-100.0)	97.9 (88.7-99.9)	>0.05	100.0 (92.5-100.0)	97.9 (88.7-99.9)	97.9 (88.7-99.9)	>0.05
>100	100.0 (96.5-100.0)	99.0 (94.8-100.0)	>0.05	100.0 (96.5-100.0)	99.0 (94.8-100.0)	99.0 (94.8-100.0)	>0.05

*SSM* sputum smear microscopy, *LAM* Urine Lipoarabinomannan strip test

*P*-value indicate significant differences between patient groups (marked with * and number to indicate comparison group) * - Significantly different (*p* ≤ 0.05) NS- Not significantly different (*p* > 0.05)

The combined sensitivity; 67.6 %, CI: 57.9–76.3 of LAM and SSM was significantly higher than that of SSM alone, being highest at ≤50 cells/μL, *p* < 0.001 (Fig. 2). The combined specificity of LAM and SSM >97 % at the three CD4 strata (Table 5) was not significantly different from that of either test alone.

## Discussion

To our knowledge, this is the first study in Zimbabwe to compare the performance characteristics of LAM with the routinely used SSM among hospitalized, HIV-infected patients with suspected TB. Our study had some interesting findings. First, employing the CRS (used as the primary analysis in this case because of sampling bias when using the MRS) we have shown that LAM is more sensitive than SSM and the combination is better than either diagnostic modality alone. Moreover, specificity was excellent. Secondly, when stratified by CD4 count, the sensitivity of LAM is highest at CD4 count ≤50 cells/μl. Similarly the sensitivity of the combined tests is highest at CD4 counts ≤50 cells/μL identifying 87 % TB-infected patients. Indeed, in Zimbabwe AIDS patients with probable opportunistic infections, such as TB, present late in the course of their disease at tertiary hospitals as reflected by the low median CD4 counts (55cells/μL) in this study. The urgent need for a rapid POC TB diagnostic tool for this group of patients cannot be over-emphasized as they may benefit from early commencement of anti-TB treatment. TB is the major cause of morbidity and mortality among HIV-infected patients [1]. SSM is the routinely used microbiological test for TB, yet it has poor sensitivity among HIV-infected patients. Our study suggests additional benefit of a rapid POC TB diagnostic tool which utilizes easily obtainable urine and produces results within 30 min.

The specificity of LAM (86 %), using MRS in our study is below the usual acceptable level of 95 % or more for acceptability of any new diagnostic test. Several reasons may be attributable to the low specificity. The reference standard culture, which uses sputum is inappropriate. It is widely known that HIV-infected patients are often sputum scarce or have paucibacillary sputa and hence do not produce “good quality” sputum, thus likely contributing to false negative culture results. To address this issue, and where sampling error is high, we opted to use the CRS that is likely more appropriate in this specific context. The specificity of LAM was significantly improved from 86 % to 98 % when using the CRS. Similarly the PPV of LAM was also greatly improved from a mere 49 % to 95 %. We agree that this introduces misclassification bias. However, this specificity is likely to be more accurate than that that obtained using MRS due to the dominating effect of sampling bias (significant possibility of false negative culture results). Nevertheless, we present both analytical strategies so the relative differences can be appreciated.

Based on the sensitivity of LAM, which was significantly higher than that of the routinely used SSM; the higher sensitivity of SSM and LAM combination than SSM alone and the high specificity and PPV of LAM when using the CRS, a combination of LAM and SSM offers a very attractive option for diagnosis of HIV-associated TB in hospitalized patients who are heavily immunosuppressed. LAM has the added advantages of using urine which is easily and rapidly obtained even from very ill patients compared to sputum. It is an easy to use POC test which can also be performed by trained nurses making it an ideal POC test [7]. It does not require electricity or any equipment, making it ideal for rural areas where the majority of patients live in resource-limited countries. Each LAM test costs less than US$3.50 [4]. Moreover, adding LAM to routine TB diagnostic work-up among HIV-infected adults with CD4 counts <100 cell/μL has been reported to be cost-effective [5].

The LAM has been evaluated globally but with widely varying sensitivity (13 % to 93 %) and specificity (87 % to 99 %) (reviewed in [11]). The variability may be attributed to the study design, study populations, hospitalized versus out-patients, HIV status, degree of immunosuppression as assessed by CD4 counts, use of fresh urine versus frozen urine, cut-off grade for positive result (grade 1 versus grade 2), and performance of the test by state certified technologist versus trained nurses etc. These variations make it difficult to compare performance characteristic of LAM across various settings. However, in the majority of the studies which stratified performance of LAM by CD4 counts, the consensus is that sensitivity of LAM was highest in patients with low CD4 counts (reviewed in [11]). Our study lends support to the growing consensus.

To our knowledge, only two other studies, one in South Africa [4] and another in Uganda [9] have evaluated LAM in hospitalized HIV-infected patients with suspected TB. The South African study [4] like our current study used both MRS and CRS for LAM grade 2 cut-point analysis. Comparison of LAM performance characteristics is not possible due to the different study designs and degree of immunosuppression. Furthermore the South African study used frozen urine whereas in the current study, spot fresh urine was used. However, the two studies (South Africa and Zimbabwe) showed that using the CRS the specificity of LAM was high (>95 %). The two studies also showed that combining LAM and SSM significantly increased the sensitivity. The Ugandan study [9] used a MRS based LAM grade 2 cut-off analysis. Based on the overlapping CI, the sensitivity in our study 61 %, CI: 49.6–71.6 was similar to that in the Ugandan study; 49 %, CI: 39.0–59.0. Conversely, the specificity of LAM was higher in the Ugandan study 97 %, CI: 92.0–99.0 versus 86 %, CI: 82.2–89.5. The differences in specificity between the two studies (Uganda and Zimbabwe) may be attributable to misclassification bias and differences in study design (case controlled versus unselected cohort design).

There are some limitations of our study. We did not perform Gene Xpert, which has been partly rolled out in several countries. Thus, comparison of LAM and Xpert was not possible. However, in most TB endemic countries globally and including Africa SSM is still the predominant diagnostic modality. Thus, our data have substantial relevance to clinical practice in TB and HIV endemic settings. Misclassification bias could have over or under estimated the accuracy of LAM. The focus of this report is to highlight the performance outcome of a combination diagnostic strategy; patient important outcomes are reported elsewhere (submitted).

## Conclusion

Among hospitalized HIV-infected patients with suspected TB, the combined sensitivity of SSM and LAM, using CRS, is significantly higher than that of SSM, especially at low CD4 counts. The specificity is greater than 95 % for either test alone or combined. Thus, LAM and SSM are complimentary tests for diagnosis of TB in HIV-infected patients. We recommend a combination of LAM and SSM for TB diagnosis in HIV-infected patients with low CD4 counts in HIV/TB co-endemic countries, where alternative methods are unavailable.
